# Supplementary material for: A large scale evaluation of TBProfiler and Mykrobe for antibiotic resistance prediction in Mycobacterium tuberculosis
Source: PeerJ. 2019 May 1;7:e6857. doi: 10.7717/peerj.6857 (PMC6500375; doi:10.7717/peerj.6857)
Supplement: Supplemental Information 1 [file peerj-07-6857-s001.pdf]

A large scale evaluation of TBProfiler and Mykrobe for antibiotic resistance prediction in *Mycobacterium tuberculosis* - supplementary materials

Pierre Mahé[1,*], Meriem El Azami[1], Philippine Barlas[1], and Maud Tournoud[1]

[1]**bioMérieux, Data Analytics Unit, Marcy l'Etoile, 69600, France**
[*]**pierre.mahe@biomerieux.com**

ABSTRACT

This document presents supplementary information for the manuscript entitled "A large scale evaluation of TBProfiler and Mykrobe for antibiotic resistance prediction in *Mycobacterium tuberculosis*".

CONTENTS

S1 `TBPofiler` and `Mykrobe` configurations **1**
S2 Lineage prediction **1**
S3 Genotyping agreement **2**
S4 `TBProfiler` and `Mykrobe` performance **2**
S5 Lineage-level `Mykrobe` performance **2**
S6 Individual marker performance **7**
S7 Analysis of high-confidence mutations reported in Miotto (2017) **7**
S8 Identification of close isolates **8**
S9 Multivariate modeling strategies **10**
References **14**

S1 TBPOFILER AND MYKROBE CONFIGURATIONS

All experiments done in this study were run using command-line versions of `TBProfiler` (version 0.3.4) and `Mykrobe` (v0.3.3-0-gc211bf2), operating on their default configurations :

- `TBProfiler` uses bwa mem (v0.7.15-r1142-dirty) with default parameters for mapping, and lofreq (v2.1.3.1) with default parameters for variant calling.
- `Mykrobe` uses mccortex with default parameters for variant calling (kmer size = 21)

The sole parameter allowed to be tuned afterwards was the frequency threshold to call a resistance allele present, as described in the main text.

S2 LINEAGE PREDICTION

Figure S1 shows the number and fractions of "mixed" lineage calls obtained by `TBProfiler` when the minimum frequency threshold to call a lineage-defining mutation is increased from 0 to 0.2. We note that introducing such a threshold allows to drastically reduce the number of mixed calls made on the 4 major lineages, but has no impact on lineages 6 (West-Africa), "BOV" and "BOV-AFRI", for which mixed calls are systematically observed. Note that this figure was computed from the 6570 samples that

	TBProfiler					Mykrobe				
	sensi	speci	precision	macro	thresh	sensi	speci	precision	macro	thresh
amikacin	92.1	87.9	71.7	90.0	0	82.6	98.5	94.7	90.5	0
	89.3-94.9	86-89.8	67.6-75.8			78.7-86.5	97.8-99.2	92.2-97.2		
capreomycin	82	94.7	83.2	88.3	0	78.3	94	80.7	86.2	0
	77.9-86.1	93.4-96	79.2-87.2			74-82.6	92.6-95.4	76.5-84.9		
ethambutol	93.3	88.8	58.7	91.0	0	87.5	93.6	70.1	90.5	0
	91.5-95.1	87.9-89.7	55.9-61.5			85.1-89.9	92.9-94.3	67.2-73		
ethionamide	85.3	60.5	56.5	72.9	0	-	-	-	-	-
	80.8-89.8	55.7-65.3	51.4-61.6			-	-	-	-	-
fluoroquinolones	89	95.8	85.9	92.4	0	85.1	97.2	89.6	91.2	0
	85.7-92.3	94.7-96.9	82.3-89.5			81.4-88.8	96.3-98.1	86.3-92.9		
isoniazid	90	95.2	86.9	92.6	0	88.5	98.3	94.8	93.4	0
	88.6-91.4	94.6-95.8	85.3-88.5			87-90	97.9-98.7	93.7-95.9		
kanamycin	91.7	95.7	89.9	93.7	0	81.7	98	94.5	89.8	0
	88.8-94.6	94.3-97.1	86.7-93.1			77.6-85.8	97-99	91.9-97.1		
pyrazinamide	61.4	91.7	75.3	76.5	0	34.4	99	93.7	66.7	0
	56.3-66.5	89.8-93.6	70.3-80.3			29.4-39.4	98.3-99.7	89.5-97.9		
rifampicin	92	91.6	72.3	91.8	0	92.4	98.3	92.8	95.3	0
	90.5-93.5	90.8-92.4	70.1-74.5			90.9-93.9	97.9-98.7	91.4-94.2		
streptomycin	78.5	88.7	73.9	83.6	0	81.9	95.5	88.2	88.7	0
	76-81	87.5-89.9	71.3-76.5			79.5-84.3	94.7-96.3	86.1-90.3		

Table S1. Overall performance of `TBProfiler` and `Mykrobe` measured in terms of sensitivity (sensi), specificity (speci), precision and macro-accuracy (macro), defined as the average between sensitivity and specificity. For each software and antibiotic, no minimum frequency threshold was considered to call a marker present. 95% confidence intervals are provided for sensitivity, specificity and precision.

were successfully processed by both `TBprofiler` and `Mykrobe` (hence excluding one sample that was successfully processed by `TBProfiler`).

Figure S2 compares the lineages inferred by `Mykrobe` and `TBProfiler`, excluding the "mixed" lineage calls made by `TBProfiler` without any minimum frequency threshold.

Figure S3 compares similarly the lineages inferred by `Mykrobe` and `TBProfiler` on the "mixed" lineage calls made by `TBProfiler` when the minimum frequency threshold to call a lineage-defining mutation is increased from 0 to 0.2. As mentioned above, we note that including a threshold allows to drastically reduce the number of "mixed" calls made for the four major lineages but not for lineages 6 (West-Africa), "BOV" and "BOV-AFRI".

S3 GENOTYPING AGREEMENT

Figure S4 compares the markers calls when the minimum frequency threshold to call a marker present is set to 0.5. The behavior is comparable to that observed in Figure 1 of the main text, indicating that the discrepancy is not due to possible ambiguities in calling markers observed at a low frequency present.

S4 `TBPROFILER` AND `MYKROBE` PERFORMANCE

Figure S5 shows the evolution of the performance obtained by `TBProfiler` and `Mykrobe` when increasing the minimum frequency threshold to call a marker present. Performance is measured in terms of macro-accuracy (the average of sensitivity and specificity) on the left-hand side, and in terms of the F1 measure (the harmonic mean of precision and recall) on the right-hand side. Results reported in the main text are chosen to maximize the macro-accuracy, for each antibiotic and each software. Results obtained without considering any threshold to call a marker present are presented in Table S1.

S5 LINEAGE-LEVEL `MYKROBE` PERFORMANCE

Table S2 shows the performance of `Mykrobe` across the 4 major lineages, as done for `TBprofiler` in Table 4 of the main text.

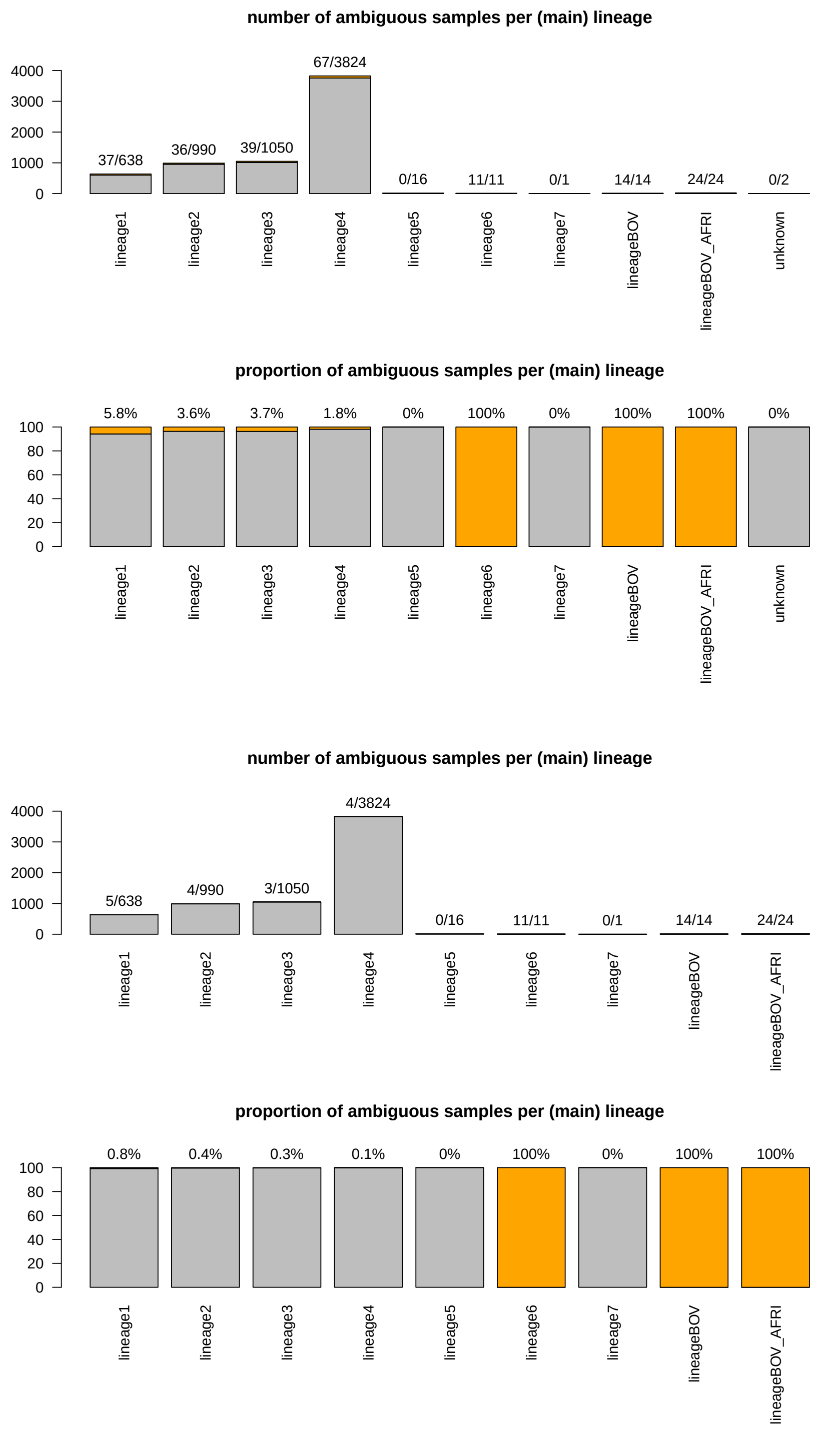

Figure S1. Number and fractions of ”mixed” lineage calls obtained by `TBProfiler` when no minimum frequency threshold to call a lineage-defining mutation is considered (top), or when it is set to 0.2 (bottom).

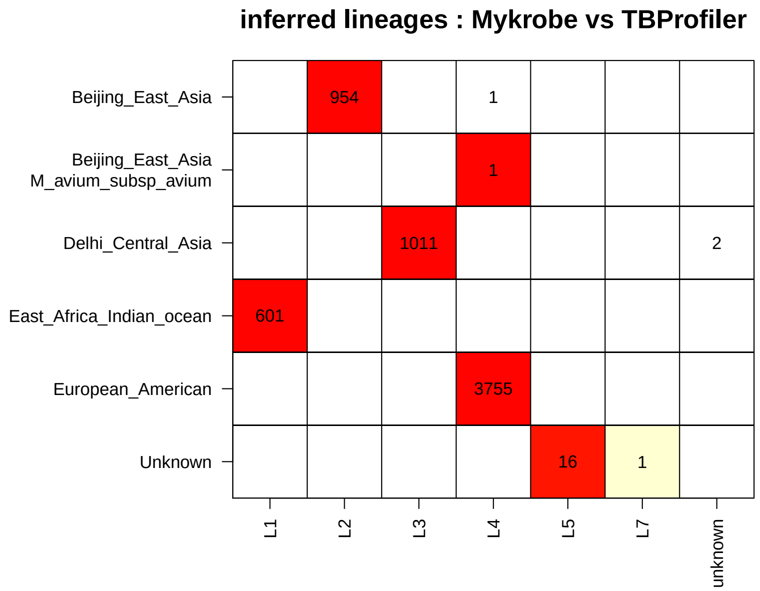

Figure S2. Comparison of lineages inferred by `Mykrobe` (in rows) and `TBProfiler` (in columns) for un-ambiguous samples.

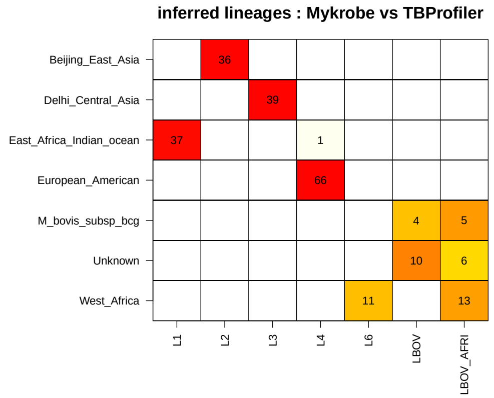

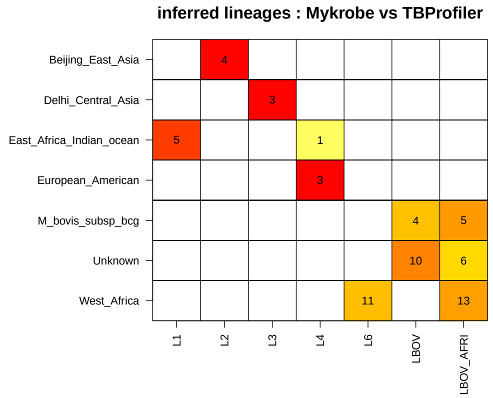

Figure S3. Comparison of lineages inferred by `Mykrobe` (in rows) and `TBProfiler` (in columns) for “mixed” samples called by `TBProfiler` when no minimum frequency threshold was considered (left), or when it was set to 0.2 (right).

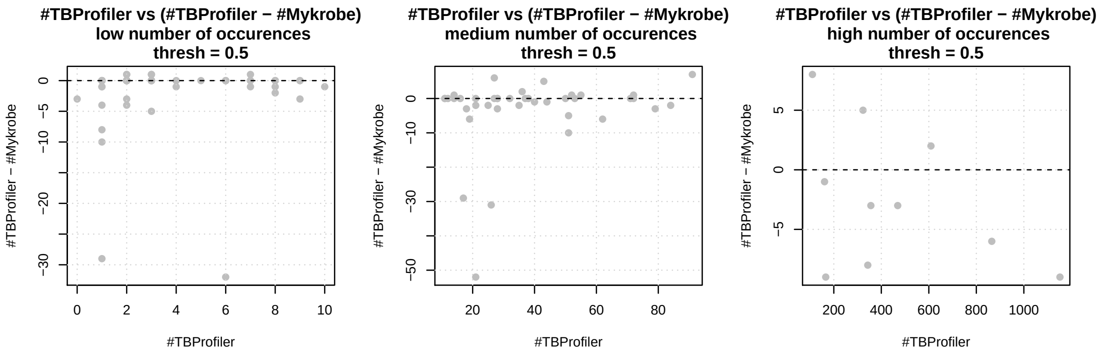

Figure S4. Comparison of the number of calls made for the 116 markers addressed by both `TBProfiler` and `Mykrobe`. Each dot corresponds to a marker and shows the difference in the number of calls made by `TBProfiler` and `Mykrobe` versus the number of calls made by `TBProfiler`. Markers are split in 3 groups, whether they are found in fewer than 10 strains (left), between 10 and 100 (middle) or more than 100 strains (right) by `TBProfiler`. The minimum frequency threshold considered to call a marker present is set to 0.5.

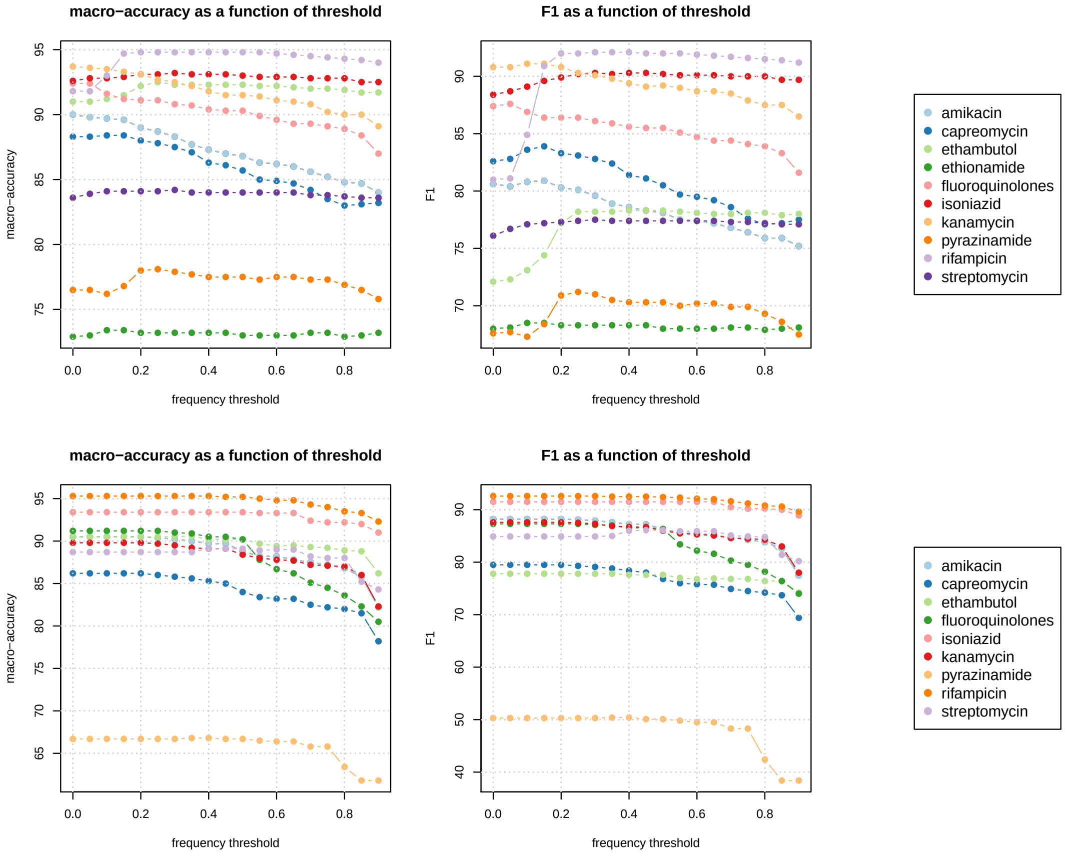

Figure S5. Overall `TBProfiler` (top) and `Mykrobe` (bottom) performance when a minimum frequency threshold to call a marker present is considered.

drug	lineage	total	S	R	%R	sensitivity	specificity	macro	precision
amikacin	global	1475	1109	366	25	82.5 (±3.9)	98.5 (±0.7)	90,5	94.7 (±2.5)
	lineage1	25	20	5	20	40 (±42.9)	100 (±0)	70	100 (±0)
	lineage2	613	369	244	40	86.9 (±4.2)	96.7 (±1.8)	91,8	94.6 (±3)
	lineage3	79	50	29	37	89.7 (±11.1)	100 (±0)	94,8	100 (±0)
	lineage4	758	670	88	12	70.5 (±9.5)	99.3 (±0.6)	84,9	92.5 (±6.6)
capreomycin	global	1430	1085	345	24	78.3 (±4.3)	94 (±1.4)	86,2	80.6 (±4.7)
	lineage1	24	23	1	4	0 (±0)	91.3 (±11.5)	45,6	0 (±NaN)
	lineage2	581	358	223	38	86.1 (±4.5)	87.4 (±3.4)	86,8	81 (±5.5)
	lineage3	75	64	11	15	90.9 (±17)	75 (±10.6)	83	38.5 (±30.2)
	lineage4	750	640	110	15	61.8 (±9.1)	99.7 (±0.4)	80,8	97.1 (±4)
ethambutol	global	5128	4371	757	15	87.5 (±2.4)	93.6 (±0.7)	90,5	70.2 (±3.5)
	lineage1	432	415	17	4	94.1 (±11.2)	97.8 (±1.4)	95,9	64 (±23.5)
	lineage2	900	465	435	48	92.9 (±2.4)	77 (±3.8)	85	79.1 (±4)
	lineage3	903	866	37	4	86.5 (±11)	96.2 (±1.3)	91,3	49.2 (±17.3)
	lineage4	2893	2625	268	9	78.4 (±4.9)	95 (±0.8)	86,7	61.4 (±6.6)
fluoroquinolones	global	1603	1248	355	22	85.1 (±3.7)	97.2 (±0.9)	91,2	89.6 (±3.4)
	lineage1	77	71	6	8	33.3 (±37.7)	100 (±0)	66,7	100 (±0)
	lineage2	427	267	160	37	86.2 (±5.3)	93.3 (±3)	89,8	88.5 (±5.3)
	lineage3	168	135	33	20	90.9 (±9.8)	99.3 (±1.4)	95,1	96.8 (±6.3)
	lineage4	931	775	156	17	84.6 (±5.7)	97.9 (±1)	91,2	89.2 (±5.3)
isoniazid	global	6398	4717	1681	26	88.6 (±1.5)	98.3 (±0.4)	93,4	94.8 (±1.1)
	lineage1	634	537	97	15	95.9 (±3.9)	98.7 (±1)	97,3	93 (±5.2)
	lineage2	981	354	627	64	95.2 (±1.7)	95.2 (±2.2)	95,2	97.2 (±1.3)
	lineage3	1050	878	172	16	89 (±4.7)	99.4 (±0.5)	94,2	96.8 (±2.8)
	lineage4	3733	2948	785	21	82.3 (±2.7)	98.2 (±0.5)	90,2	92.6 (±2)
kanamycin	global	1152	815	337	29	81.6 (±4.1)	98 (±1)	89,8	94.5 (±2.7)
	lineage1	26	20	6	23	33.3 (±37.7)	100 (±0)	66,7	100 (±0)
	lineage2	397	200	197	50	86.8 (±4.7)	96 (±2.7)	91,4	95.5 (±3.1)
	lineage3	73	45	28	38	89.3 (±11.4)	100 (±0)	94,7	100 (±0)
	lineage4	656	550	106	16	72.6 (±8.5)	98.5 (±1)	85,5	90.6 (±6.5)
pyrazinamide	global	1162	821	341	29	34 (±5)	99 (±0.7)	66,5	93.5 (±4.5)
	lineage1	132	125	7	5	14.3 (±25.9)	98.4 (±2.2)	56,4	33.3 (±92.4)
	lineage2	246	82	164	67	37.2 (±7.4)	97.6 (±3.3)	67,4	96.8 (±4.4)
	lineage3	147	116	31	21	9.7 (±10.4)	100 (±0)	54,9	100 (±0)
	lineage4	637	498	139	22	36.7 (±8)	99.2 (±0.8)	68	92.7 (±7.1)
rifampicin	global	6361	5127	1234	19	92.4 (±1.5)	98.3 (±0.4)	95,3	92.9 (±1.5)
	lineage1	635	607	28	4	89.3 (±11.4)	97.9 (±1.1)	93,6	65.8 (±18.6)
	lineage2	966	363	603	62	96.4 (±1.5)	96.1 (±2)	96,2	97.6 (±1.2)
	lineage3	1049	967	82	8	85.4 (±7.6)	99.2 (±0.6)	92,3	89.7 (±7.1)
	lineage4	3711	3190	521	14	89.1 (±2.7)	98.4 (±0.4)	93,8	89.9 (±2.7)
streptomycin	global	3462	2453	1009	29	81.2 (±2.4)	96.9 (±0.7)	89,1	91.6 (±1.9)
	lineage1	233	209	24	10	75 (±17.3)	92.8 (±3.5)	83,9	54.5 (±23)
	lineage2	792	275	517	65	97.9 (±1.2)	93.1 (±3)	95,5	96.4 (±1.6)
	lineage3	297	253	44	15	59.1 (±14.5)	98 (±1.7)	78,5	83.9 (±14.1)
	lineage4	2140	1716	424	20	63.4 (±4.6)	97.9 (±0.7)	80,7	88.2 (±3.9)

Table S2. `Mykrobe` performance across the 4 major lineages. Figures between brackets correspond to 95% confidence intervals. Shown in grey are the lineages with less than around 100 strains. Shown in orange and green are the lineages where the macro accuracy is lesser or greater than the global one by more than 5 points. These thresholds were set arbitrarily.

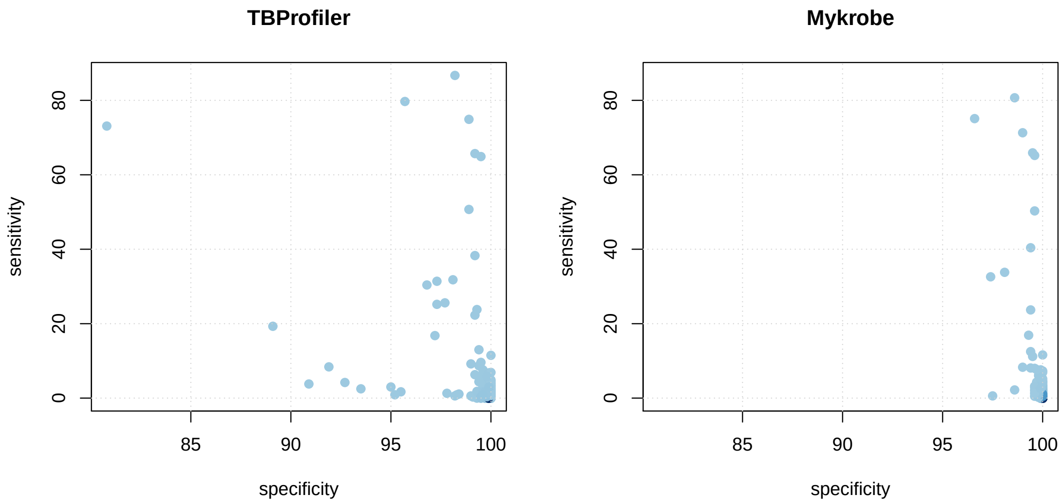

Figure S6. Individual markers performance, considering all antibiotics altogether. Left : `TBProfiler`; Right : `Mykrobe`.

S6 INDIVIDUAL MARKER PERFORMANCE

Figure S6 shows the individual markers performance globally, i.e., considering all drugs altogether. Each corresponds to a given mutation in the `TBProfiler` (left) and `Mykrobe` (right) catalog and represents its sensitivity as a function of its specificity. We globally note that a limited number of mutations have a large sensitivity.

Table S3 provides the individual amikacin markers performance. Interestingly, we note that the markers detected are not strictly the same, or not always detected in the same number of strains:

- `Mykrobe` solely detected 7 mutations in *rss*, while `TBProfiler` also detected 7 mutations in *rss* and 2 mutations in *eis*.
- both softwares detected the `A>G` mutation at position 1401 in *rss*, but in 339 and 311 strains with `TBProfiler` and `Mykrobe`, respectively. `Mykrobe` however detected the mutations `A>C` and `A>T` at the same position in 10 and 9 strains respectively, which are not detected by `TBProfiler`.
- `TBProfiler` detected mutations at position 514 and 517 in a relatively significant number of strains (147 and 22 respectively), which are not considered by `Mykrobe`.

The large gap observed in terms of sensitivity (82.6% for `Mykrobe` vs 92.1% for `TBProfiler`) is probably mostly due to the mutation in *rss* at position 514. Choosing to integrate or not this mutation in one's catalogue amounts to choosing to favour sensitivity over specificity, and is therefore a matter of choice (or of target performances one wants to achieve).

S7 ANALYSIS OF HIGH-CONFIDENCE MUTATIONS REPORTED IN MIOTTO (2017)

As discussed in the main text, a limitation of this study, as any study lead in a similar setting, lies in the fact that phenotypic antimicrobial susceptibility testing is an imperfect gold-standard (Brennan-Krohn et al., 2017). A list of high-confidence mutations was however recently proposed by Miotto et al. (2017). It is considered trustworthy enough by the WHO to correct phenotypes determined phenotypically: isolates harboring these mutations are systematically considered as resistant in World Health Organization (2018), even if they were identified as susceptible by phenotypic testing. We therefore aimed to evaluate how many isolates harboring these mutations were reported as phenotypically susceptible in this dataset.

For this purpose, we considered the high-confident mutations reported in Miotto et al. (2017) with significant associations up to p-values correction (shown in bold in Table 3 of this paper). We did not

id	coverage	sensi	speci	precision
rrs_1158G>T	1	0	99,9	0
eis-Rv2417c_-10C>G	2	0	99,8	0
rrs_1402C>T	5	1,1	99,9	80
rrs_1402C>A	8	0	99,3	0
rrs_1484G>T	13	1,4	99,3	38,5
rrs_517C>T	22	1,1	98,4	18,2
eis-Rv2417c_-10C>T	66	3	95	16,7
rrs_514A>C	147	30,4	96,8	76,2
rrs_1401A>G	339	86,7	98,2	94,1
joint	473	92,1	87,9	71,7

id	coverage	sensi	speci	precision
rrs_C1402A	1	0	99,9	0
rrs_G1484C	1	0,3	100	100
rrs_C1402T	3	0,5	99,9	66,7
rrs_G1484T	6	1,4	99,9	83,3
rrs_A1401T	9	2,5	100	100
rrs_A1401C	10	2,7	100	100
rrs_A1401G	311	80,7	98,6	95,2
joint	320	82,6	98,5	94,7

Table S3. Comparison of the individual amikacin markers performance captured by `TBProfiler` (left) and `Mykrobe` (right).

however consider mutations leading to frameshits or premature stop codons as they were not readily available in the results provided by `TBprofiler` nor `Mykrobe`, and considered the significant mutation of moderate confidence for ethionamide, as no significant high-confidence mutation was available for this drug. Altogether, this provided us with a list of 94 mutations.

Table S4 summarizes the results obtained. We first noted that not all mutations could be found in the genotype matrices built from both softwares, which may be due to the fact that these mutations were never observed on this dataset, or that they were not part of their catalogs of mutations in the first place. A greater number of mutations could often be retrieved using `TBProfiler`, which is consistent with the fact that it considers a larger list of mutations. We then noted that less than 2% of susceptible strains harbored at least one of these mutations for most drugs. This was the case of all drugs except fluoroquinolones and capreomycin, where this proportion rose to 3-4%, and ethionamide, where it rose to 19%, most probably due to the fact that the confidence in the underlying mutation is moderate. These figures were obtained considering a minimum allele frequency threshold set to 0.2 to call a marker present with `TBProfiler`. When no minimum frequency threshold was considered, these figures increased significantly in some cases (e.g., by almost 5 points for pyrazinamide and 1 point for amikacin). Interestingly, Table S5 provides the predictive performance, defined in terms of sensitivity and specificity, of `TBProfiler` and `Mykrobe` operating on this set of high-confidence mutations. Unsurprisingly, focusing on such highly specific mutations leads to highly specific models, but has a price in terms of sensitivity, which can be quite high (e.g., 20 points for isoniazid).

A table providing these results on a marker basis, together with the correspondence between the mutations identifiers in Miotto et al. (2017), `TBProfiler` and `Mykrobe` is available upon request to the authors.

S8 IDENTIFICATION OF CLOSE ISOLATES

As discussed in the main text, the presence of groups of highly-related isolates (e.g., coming from an outbreak) may bias the predictive performance estimation. A standard way to circumvent this issue would amount to identifying such groups of close isolates using a SNP-based distance criterion defined at the whole-genome level, and to pick one isolate per group. This would require however to have access to the assembled genomes of the isolates, which is not provided by `TBProfiler` nor `Mykrobe`. Without delving into an extensive genome assembly study, we aimed to assess the presence of such groups of close isolates by quantifying their distance in terms of SNPs observed within the resistance loci addressed by `TBProfiler`. Indeed, since `TBProfiler` reports any "novel" mutation found within these loci, we can readily compute a SNP-based distance criterion restricted to this set of loci.

To identify such groups of (putatively) close isolates, we therefore proceeded as follows :

- we characterized an isolate from the entire list of "novel" mutations reported by `TBProfiler`. Importantly, we chose not to introduce any minimum frequency threshold to call these mutations present. While we risk to call false-positive mutations doing so, considering a minimum frequency threshold reduces the number of mutations detected, hence has the opposite risk of reducing the resolution of the analysis. We empirically observed that considering such a minimum frequency

	Number of mutations			Phenotypes		Putative FN		
	Miotto	TBP	MYK	R	S	TBP - thresh = 0	TBP -thresh = 0.2	MYK
amikacin	2	2	2	367	1108	28 (2.53)	17 (1.53)	16 (1.44)
capreomycin	4	4	3	345	1084	55 (5.07)	40 (3.69)	38 (3.51)
ethionamide	1	1	0	237	395	76 (19.24)	74 (18.73)	–
fluoroquinolones	10	9	7	355	1249	40 (3.2)	37 (2.96)	34 (2.72)
isoniazid	2	2	2	1692	4770	37 (0.78)	24 (0.5)	22 (0.46)
kanamycin	3	3	1	337	815	22 (2.7)	19 (2.33)	8 (0.98)
pyrazinamide	49	37	16	346	840	44 (5.24)	4 (0.48)	3 (0.36)
rifampicin	18	14	16	1236	5187	35 (0.67)	29 (0.56)	30 (0.58)
streptomycin	5	5	4	1014	2490	49 (1.97)	29 (1.16)	28 (1.12)

Table S4. Analysis of high-confidence Miotto et al. (2017) mutations. Number of mutations : Miotto = number of high-confidence mutations mutations reported in Miotto et al. (2017) (see Section S7 for details) ; TBP/MYK = number of mutations found in genotype matrices obtained by `TBProfiler` and `Mykrobe` in this study. Phenotypes: number of R and S phenotypes available. Putative FN = number and rate (%) of putative False Negative (FN) phenotypes (strains harboring at least one high-confidence mutation reported as susceptible), based on `TBProfiler` or `Mykrobe` results. Two configurations were considered for `TBProfiler`: using a minimum allele frequency of 0.2 to call a marker present, and no minimum threshold. Such a threshold had no influence on `Mykrobe` results.

	TBP - thresh = 0		TBP - thresh = 0.2		MYK	
	sensi	speci	sensi	speci	sensi	speci
amikacin	88,28	97,47	84,74	98,47	82,02	98,56
capreomycin	82,03	94,93	79,71	96,31	77,97	96,49
ethionamide	73,42	80,76	73	81,27	–	–
fluoroquinolones	85,92	96,8	83,1	97,04	83,94	97,28
isoniazid	67,38	99,22	67,08	99,5	67,2	99,54
kanamycin	86,35	97,3	83,09	97,67	8,01	99,02
pyrazinamide	30,92	94,76	29,19	99,52	25,43	99,64
rifampicin	86,57	99,33	86	99,44	86,97	99,42
streptomycin	71,99	98,03	71,1	98,84	70,91	98,88

Table S5. Analysis of high-confidence Miotto et al. (2017) mutations. Sensitivity / specificity obtained by `TBProfiler` and `Mykrobe` operating on the list of high-confidence mutations reported in Table S4.

threshold had the effect of drastically increasing the fraction of closely-related isolates, which lead us to lowering this threshold down to zero.

- we then simply define the distance between two isolates as the number of SNPs by which they differ.
- we finally apply a standard hierarchical clustering process based on the distance matrix obtained, and "cut" the resulting dendrogram at various levels to identify groups (or "clusters") of close isolates. Cutting the dendrogram can directly be interpret in terms of (maximum) withing-group SNP distance: members of the clusters identified differ by at most a number of SNPs equal to the value considered to cut the dendrogram.

This criterion is probably much less sensitive than considering a similar distance defined at the whole-genome level. It is also harder to interpret, and defining the appropriate level at which to cut the dendrogram is difficult.

Figure S7 summarizes the results of this analysis when the dendrogram is cut at the level of 0 and 1 SNP (top and bottom, respectively). At the level of 0 SNP, isolates are considered as close if they harbor exactly the same set of mutations. Note that these samples are nevertheless not identical: despite the fact that they have the same set of mutations, these mutations were not detected with the same allele frequency by `TBProfiler` (in which case the raw FASTQ files would be identical, which we checked beforehand).

Among the 6571 samples that were successfully processed by `TBProfiler`, 6338 had a unique set of mutations (i.e., no other sample could be found at a distance of 0 SNP). The remaining 233 samples were clustered in 64 groups. 75% of these clusters involving 2 or 3 samples (37 clusters of size 2 and 11 clusters of size 3), and the 4 larger clusters involved 10 to 21 isolates. Figure S8 provides a representation of the distance matrix and dendrogram restricted to these 233 samples, which allows to note that the majority of these samples originate from the Mykrobe study (Bradley et al., 2015). Tolerating a distance of 1 SNP to define isolates as close leads naturally to clustering a greater number of samples together. More precisely, among the 6571 samples, 6092 are still considered as sufficiently distant from any other sample, while the 479 remaining ones are gathered in 162 clusters (Figure S7, bottom). The majority of these clusters also involved 2 or 3 isolates, and the 4 major clusters identified previously grow to include 11 to 26 isolates.

Depending on the number of SNPs considered to claim isolates as sufficiently close, this analysis therefore suggested that 6402 (6338+64) or 6254 (6092+162) groups of sufficiently distinct isolates can be found among the 6571 samples, which therefore constitutes around 97.5% or 95% of the dataset. We emphasize however that defining thresholds on SNP-based distance matrices defined from such a limited number of loci is arbitrary and hazardous, hence that these results must be interpreted with caution. They nevertheless indicate that some groups of close isolates are probably indeed present in the dataset, but suggest that this issue is marginal. An interesting perspective of this work could amount to consolidating this analysis after a preliminary step of genome assembly, in order ultimately to refine the estimation of the predictive performances after the exclusion of such close isolates.

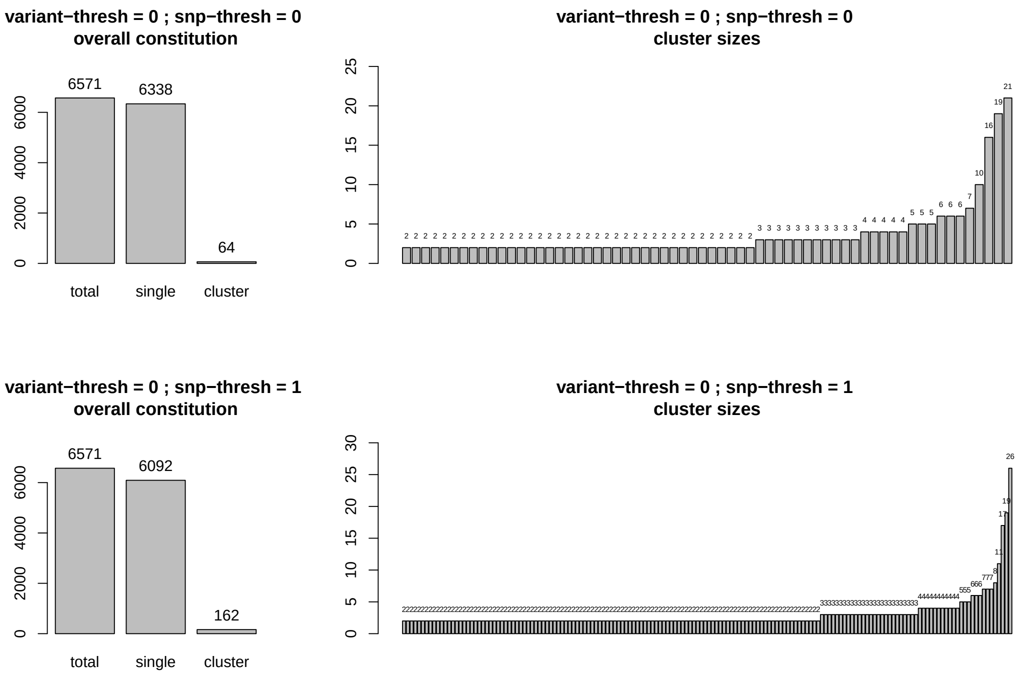

Figure S7. Number and size of clusters found when the dendogram built from the SNP-based distance matrix derived from the "novel" mutations identified by `TBProfiler` is cut at the level of 0 SNP (top) or 1 SNP (bottom). Left : total number of samples successfully processed by `TBProfiler` vs number of isolates considered sufficiently distant from any other isolate ("single") and number of clusters found ("cluster"). Right : sizes of the clusters found.

S9 MULTIVARIATE MODELING STRATEGIES

Figure S9 shows the ROC curves obtained by multivariate machine learning modeling strategies for the 6 antibiotics not shown on the main text, namely capreomycin, ethambutol, ethionamide, fluoroquinolones,

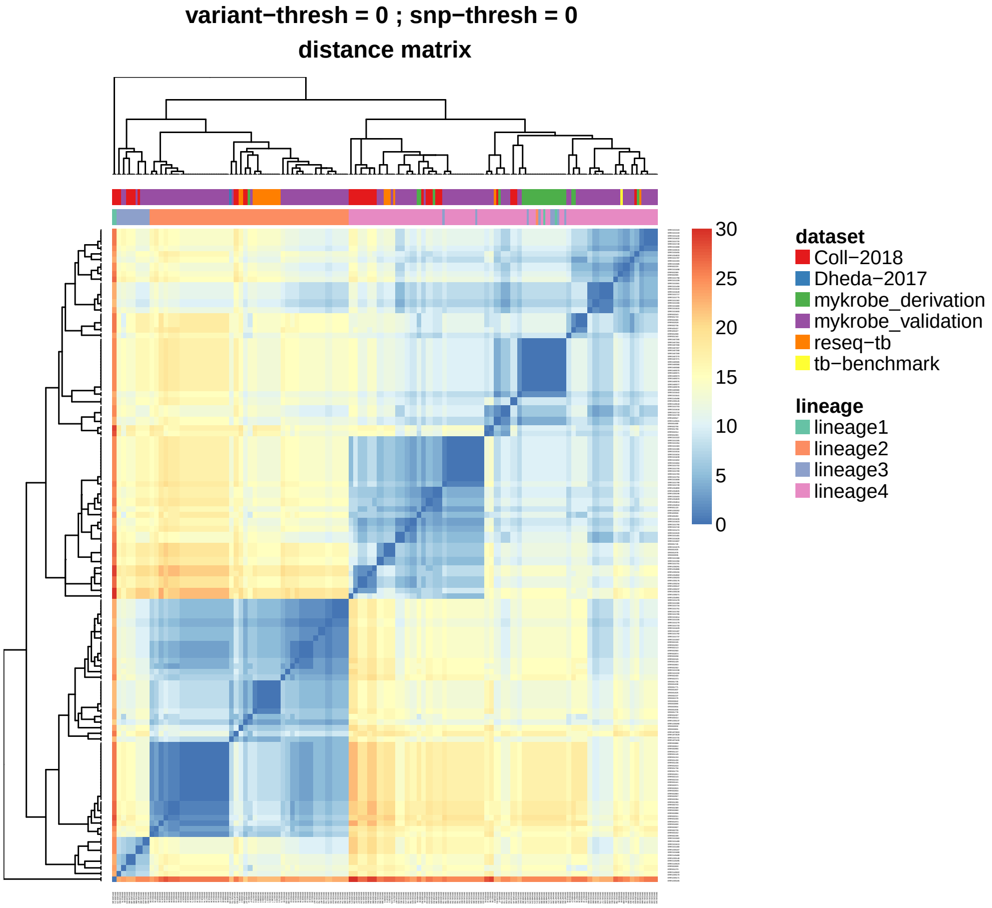

Figure S8. SNP-based distance matrix computed from the "novel" mutations identified by `TBProfiler` restricted to the samples having a non-unique set of mutations. These samples correspond to the 233 samples entering a cluster when the dendrogram built from the SNP-based distance matrix was cut at the level of 0 SNP (see Figure S7).

isoniazid and kanamycin.

Figure S10 compares the performance obtained by Lasso-penalized logistic-regression models operating from the original set of markers detected by TBP and the inclusion of novel mutations discovered during the genotyping process.

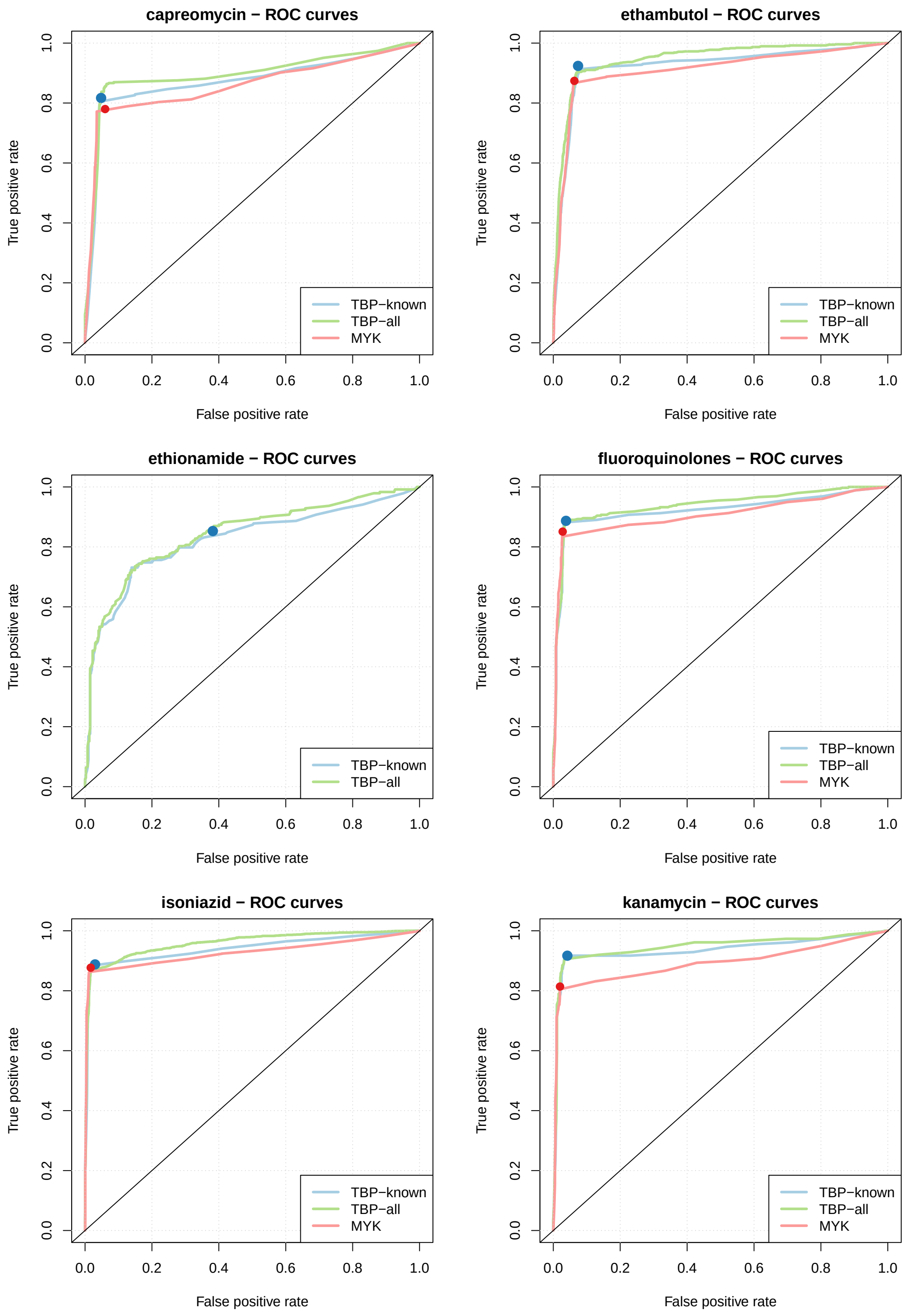

Figure S9. Illustration of ROC curves obtained by L1-penalized logistic regression using `TBProfiler` and `Mykrobe` markers. The "TBP-known" model is built using the `TBProfiler` known markers only, the "TBP-all" model using the known and the novel mutations identified by `TBProfiler`, and the "MYK" model using the `Mykrobe` markers. The red and blue dots represent performances respectively obtained by `TBProfiler` and `Mykrobe` softwares under the same cross-validation process.

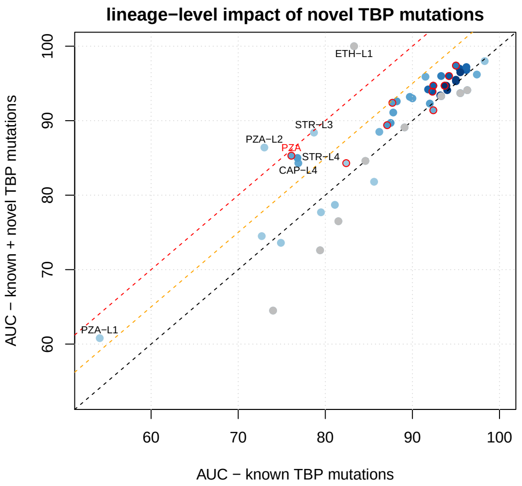

Figure S10. Lineage-level analysis of performance brought by novel `tbprofiler` mutations. Each point corresponds to the AUC measured for a given antibiotic within a given lineage. On the *x*-axis: performance of the model considering known `tbprofiler` mutations only. On the *y*-axis: performance of the model considering in addition the novel mutations identified by `tbprofiler`. Points circled in red correspond to the global performance (i.e., on the four main lineages). Dashed orange and red lines represent an improvement of 5 and 10 points, respectively. Drug/lineage configurations with more than 5 point improvement are indicated in the figure. Grey dots correspond to drug/lineage configurations for which less than 100 strains with phenotypes are available.

REFERENCES

Bradley, P., Gordon, N. C., Walker, T. M., Dunn, L., Heys, S., Huang, B., Earle, S., Pankhurst, L. J., Anson, L., de Cesare, M., Piazza, P., Votintseva, A. A., Golubchik, T., Wilson, D. J., Wyllie, D. H., Diel, R., Niemann, S., Feuerriegel, S., Kohl, T. A., Ismail, N., Omar, S. V., Smith, E. G., Buck, D., McVean, G., Walker, A. S., Peto, T., Crook, D., and Iqbal, Z. (2015). Rapid antibiotic-resistance predictions from genome sequence data for *Staphylococcus aureus* and *Mycobacterium tuberculosis*. *Nature Communications*, 6:10063.

Brennan-Krohn, T., Smith, K. P., and Kirby, J. E. (2017). The poisoned well: Enhancing the predictive value of antimicrobial susceptibility testing in the era of multidrug resistance. *Journal of Clinical Microbiology*, 55(8):2304–2308.

Miotto, P., Tessema, B., Tagliani, E., Chindelevitch, L., Starks, A. M., Emerson, C., Hanna, D., Kim, P. S., Liwski, R., Zignol, M., Gilpin, C., Niemann, S., Denkinger, C. M., Fleming, J., Warren, R. M., Crook, D., Posey, J., Gagneux, S., Hoffner, S., Rodrigues, C., Comas, I., Engelthaler, D. M., Murray, M., Alland, D., Rigouts, L., Lange, C., Dheda, K., Hasan, R., Ranganathan, U. D. K., McNerney, R., Ezewudo, M., Cirillo, D. M., Schito, M., Köser, C. U., and Rodwell, T. C. (2017). A standardised method for interpreting the association between mutations and phenotypic drug resistance in *Mycobacterium tuberculosis*. *European Respiratory Journal*, 50(6).

World Health Organization (2018). Global tuberculosis report 2018.
